# What Psychological Factors Make Individuals Believe They Are Infected by Coronavirus 2019?

**DOI:** 10.3389/fpsyg.2021.667722

**Published:** 2021-04-22

**Authors:** Hojjat Daniali, Magne Arve Flaten

**Affiliations:** Department of Psychology, Norwegian University of Science and Technology, Trondheim, Norway

**Keywords:** coronavirus disease 2019, expectations, health anxiety, personality dimensions, conscientiousness, nocebo effects

## Abstract

**Background:** We previously showed, by means of an online-based survey, that the belief of being infected by coronavirus disease 2019 (COVID-19) acted as a nocebo and predicted higher perception of symptoms similar to COVID-19 symptoms. However, there is little known about the psychological mechanisms that give rise to beliefs such as certainty of being infected by COVID-19, and this was investigated in the present study.

**Objective:** Using the same data from the previous online survey with the same research team, we further investigated whether certainty of being infected by COVID-19 is associated with age, sex, health anxiety, and/or personality traits.

**Methods:** Respondents (*N* = 375) filled out an online survey with 57 questions about symptoms similar to COVID-19, certainty of being infected by COVID-19, anxiety, stress, health anxiety, and personality dimensions (based on the five-factor model of personality).

**Results:** Higher levels of conscientiousness and health anxiety were independently associated with certainty of being infected by COVID-19. The model predicted 29% of the variance in certainty of being infected by COVID-19.

**Conclusion:** Being conscientious and worried about health issues were associated with the belief of being infected by COVID-19. Such finding may have implications for health care personnel who provide COVID-19 testing or consulting services to general population, as individuals high in these traits may over-report COVID-like symptoms. Theoretically, these findings point to psychological factors that may increase nocebo and possibly placebo effects. Clinically, the findings suggest that individuals high in conscientiousness and health anxiety may be more likely to over-report their bodily experiences.

## Introduction

Nocebos are medically inactive substances or procedures that make the individual expect unpleasant outcomes (Mitsikostas et al., 2020). The underlying mechanisms for nocebo effects are expectations and former experiences with treatments. Nocebo effects lower treatment outcomes, increase reporting of side-effects of treatments, and may impose extra pressure on the health care system (Petrie and Rief, 2019).

The recent global health threat, Coronavirus disease 2019 (COVID-19) is a highly infectious respiratory disease that is being widely spread across the globe (World Health Organization, 2020), affecting more than 120 million people so far (Worldometer, 2021). Symptoms of COVID-19 include dry cough, shortness of breath, fatigue, myalgia, and fever that are highly variable across individuals in terms of the severity and the course of the disease. Infected individuals mostly fall in a wide spectrum between experiencing no symptoms *via* mild to moderate symptoms that require no special treatment for recovery, to severe life-threatening respiratory symptoms (He et al., 2020; Moghadas et al., 2020). COVID-19 symptoms resemble symptoms of conventional seasonal influenza (Fauci et al., 2020). Thus, following the experience of symptoms similar to COVID-19 symptoms (COVID-like symptoms), the individual may suspect being infected by COVID-19, and may report the symptoms as COVID-19 symptoms.

Daniali and Flaten (2021, under review) showed that reports of COVID-like symptoms were independently predicted by both a cognitive factor, i.e., certainty of being infected by COVID-19, and by anxiety. The present study focuses on the factors that are associated with the belief of being infected by COVID-19. Very few studies have investigated the psychological factors that underlie the formation of beliefs or expectations that are central in the elicitation of nocebo and placebo effects (Flaten et al., 2013). Therefore, using the same data as the previous study (Daniali and Flaten, 2021, under review), we investigated whether sex, age, personality factors, or health anxiety predicted certainty of being infected by COVID-19.

Health anxiety refers to worrying about health in an inappropriate and exaggerated way. This construct has been shown to increase (e.g., Taylor and Asmundson, 2004) and spread during public pandemics (e.g., H1N1 influenza: Bish and Michie, 2010; Ebola: Blakey et al., 2015; SARS: Xie et al., 2011), with relations to higher COVID-19 anxiety (e.g., Lee, 2020; Son et al., 2020; Wang et al., 2020). Thus, it is likely that higher health-related concerns elevate the certainty of being infected by COVID-19.

Personality characteristics, defined as individual differences in traits and patterns of thinking, feeling, and behaving (McCrae and Costa, 2003), have been shown to modulate health-related behaviors. According to the five-factor model of personality (FFM; Costa and McCrae, 1992), individual traits are categorized into five major personality dimensions: “extroversion, agreeableness, conscientiousness, neuroticism and openness” (McCrae and Costa, 1997). Specifically, neuroticism and conscientiousness are shown to be the most pertinent personality traits in prediction of health-related behaviors. For instance, individuals scoring higher in conscientiousness, have lower health risk behaviors (e.g., Hakulinen et al., 2015); and individuals higher in neuroticism display more health risk behaviors such as smoking (e.g., Hakulinen et al., 2015). Thus, personality traits, specifically neuroticism and conscientiousness, may contribute to the belief of certainty of being infected by COVID-19.

It is shown that females report higher nocebo effects compared to males (Vambheim and Flaten, 2017), and report more COVID-like symptoms (Daniali and Flaten, 2021, under review) so it is logical to assume that females have stronger beliefs of being infected by COVID-19 than males. Moreover, as the elderly are at a higher risk of adverse consequences of COVID-19, elderly individuals could be more likely to develop beliefs about being infected by COVID-19.

Taken together, we assumed a model in which age, sex, health anxiety and personality factors, specifically neuroticism, and conscientiousness, predict the belief of certainty of being infected by COVID-19.

## Materials and Methods

### Respondents

The present study used the data from a previous study (Daniali and Flaten, 2021, under review).

Briefly, the sample included 135 males (*Min_age_* = 17, *Max_age_* = 79, *Range* (lowest minus highest) = 62, *M_age_* = 33.18, *SD* = 12.06) and 279 females (*Min_age_* = 16, *Max_age_* = 71, *Range* = 55, *M_age_* = 32.95, *SD* = 10.40) and three as “other gender” who filled out an online survey. Other gender respondents and those who were tested (regardless of the results) for COVID-19 (9.4%, *N* = 39) were excluded which resulted in a total of 375 participants (*Min_age_* = 16, *Max_age_* = 79, *Range* = 63, *M_age_* = 32.72, *SD* = 10.90) including 126 males (*Min_age_* = 17, *Max_age_* = 79, *Range* = 62, *M_age_* = 32.88, *SD* = 12.13) and 249 females (*Min_age_* = 16, *Max_age_* = 67, *Range* = 51, *M_age_* = 32.64, *SD* = 10.24).

### Measures

*Demographic questions*: Using four items, participants specified their sex, age, education level, and whether they have tested for COVID-19.

*COVID-19 certainty*: Certainty of being infected by COVID-19 was rated on a five-point Likert single item starting from “Sure not infected” that was anchored to (0) and ending with “Certain that infected” anchored to (4).

*COVID-19 symptoms*: Using 10 five-point Likert questions starting from (0) anchored to “None,” to (4) that was anchored to “Severe,” participants rated the severity of a set of symptoms similar to symptoms of COVID-19 during the last 2 months before participating in the survey. Participants rated the severity of their “myalgia (bodily pain),” “fatigue,” “cough,” “dry cough,” “sore throat,” “difficulty in breathing,” “fever,” “persistent fever,” and “headache.” The items have been previously used to measure symptoms of COVID-19 in the general population (Wang et al., 2020). For more information about the severity of symptoms similar to COVID-19, the reader is referred to the previous study (Daniali and Flaten, 2021, under review).

*Personality dimensions*: Participants filled out the Big Five Inventory short version (BFI-10) (John et al., 1991). Using 10 statements that are rated on a five-point Likert scale ranging from “1” to “5,” BFI measures the five-factor model of personality that includes “extroversion, agreeableness, conscientiousness, neuroticism and openness.” Each personality dimension is assessed by two statements. BFI-10 is a reliable and valid personality inventory with satisfactory psychometric properties (e.g., Hahn et al., 2012).

*Health anxiety*: Health anxiety was assessed by the Short Health Anxiety Inventory (SHAI; Salkovskis et al., 2002) including 18 items measuring health anxiety independent of physical health status. Each item provides four different statements about a health-related worry that are scored from (0) to (3). Items assess worrying about health, awareness of bodily sensations or changes, and feared consequences of having an illness. The SHAI has acceptable psychometric properties (e.g., Salkovskis et al., 2002). The last four items of SHAI assess “negative consequences” and are separately scored (Salkovskis et al., 2002). Therefore, in this study only the sum of the first 14 items was used as the SHAI scores. The internal consistency for the first 14 items of SHAI in the present study was 0.85.

Stress and anxiety were also assessed with 14 items, but as they overlapped with health anxiety, the data related to stress and anxiety is not analyzed in this study. In total, the survey consisted of 57 items.

### Procedure

With a focus on the general population, the online survey was first shared with the Norwegian University of Science and Technology (NTNU) students and staff *via* the intranet and then was shared in other social media such as Facebook, Instagram, Twitter, and LinkedIn from 2nd of May 2020, to the 3rd of August. Respondents were informed that the study is conducted unanimously and that they must agree to the state of consent before taking part. Both healthy participants and COVID-19 patients could participate, as the study was aimed to test the hypotheses in the general population. The study was introduced to participants as an investigation on “the effects of psychological factors on symptoms related to COVID-19,” that seeks individuals’ thoughts, personality, negative emotions such as stress and anxiety, and physical symptoms like COVID-19. Participants had to be able to comprehend English. The study holds approval from the Regional committee for medical and health research ethics, Norway (REK; project number: 142652), and the Norwegian Center for Research Data (NSD; project number: 605612).

### Statistical Analyses

The data were analyzed by SPSS software 27 (SPSS, Inc., Chicago, Illinois). First, data was screened for outliers and missing values. Next, descriptive statistics were analyzed by means of Means, *SD*s, Maxes, Mines, and the percentile distribution of the certainty of being infected by COVID-19. Next, the correlation between variables was investigated using two-tailed *Pearson* correlation. Followingly, multiple regression assumptions were checked, and issues were resolved. Finally, due to the non-normality of the residuals (see Data Screening and Preparation), a multiple regression analysis with Huber-white heteroscedasticity-consistent (HC) SEs was run to test the proposed model including variables age, sex, health anxiety, and personality factors, as the independent variables (IVs) to predict the certainty of being infected by COVID-19. Moreover, the internal consistency for 14 health anxiety items was calculated using Cronbach alpha.

### Data Screening and Preparation

No outliers or missing values were detected. To test the normality, heteroscedasticity, homoscedasticity, multicollinearity, and linearity of the variables, a multiple regression was run. Certainty of being infected by COVID-19, as the dependent variable (DV), was regressed on age, sex, health anxiety, and five personality factors (extraversion, agreeableness, openness, neuroticism, and conscientiousness) as the independent variables (IVs). The results indicated that health anxiety (*B* = 0.04, *S.E* = 0.009, *β* = 0.24, *p* = 0.0001) and conscientiousness (*B* = 0.08, *S.E* = 0.03, *β* = 0.16, *p* = 0.003) predicted certainty of being infected by COVID-19. However, the histogram plot showed that certainty was not normally distributed, and the Breusch-Pagan test supported the unreliability of the residuals (*X* ^2^ = 13.72, *p* = 0.0002; Breusch and Pagan, 1979). Then, following the suggestions by Hayes and Cai (2007), HC SEs were implemented to control for the heteroscedastic residuals. To test the homoscedasticity assumption, a multiple regression with homoscedasticity-robust SEs was conducted and the results indicated that health anxiety and conscientiousness still significantly predicted the DV. Moreover, the variance inflation (VIF) and tolerance of IVs fell in the acceptable range (tolerance > 0.20; VIF ≤ 10). Lastly, the linearity assumption was met as evidenced by the scatter plot.

Before conducting the multiple regression analysis, the data for personality dimensions were first centered by subtracting the raw data for each individual from the total mean, as the raw data for the personality dimensions did not include a zero value. Then, the centered data were included into the regression model using the “enter” method.

## Results

### Descriptive Statistics

The means of the study variables are presented in Table 1. With respecting to certainty of being infected by COVID-19, 26.9% of participants reported “sure *not infected*,” 45.6% reported “*probably not infected,*” 17.9% were “uncertain,” 7.5% were “*quite certain*” and 2.1% were “*certain*” of being infected by COVID-19 (Table 2).

**Table 1:** Descriptive statistics of the included variables.

Sex	Certainty	Age	H-Anxiety	Ep	Ap	Cp	Np	Op
Females M; SD *N* = 249 (Min; Max)	1.08; 0.89 (0; 4)	32.64; 10.24 (16; 67)	11.38; 5.44 (1; 30)	6.22; 1.97 (2; 10)	7.46; 1.58 (3; 10)	6.98; 1.89 (2; 10)	6.34; 2.19 (2; 10)	6.98; 1.89 (3; 10)
Males M; SD *N* = 126 (Min; Max)	1.21; 1.07 (0; 4)	32.88; 12.13 (17; 79)	11.15; 6.35 (0; 28)	5.75; 1.91 (2; 10)	7.10; 1.75 (2; 10)	7.11; 1.77 (2; 10)	5.48; 2.26 (2; 10)	6.74; 1.68 (2; 10)
Total M; SD *N* = 375 (Min; Max)	1.13; 0.96 (0; 4)	32.72; 10.90 (16; 79)	11.31; 5.76 (0; 30)	6.07; 1.96 (2; 10)	7.35; 1.64 (2; 10)	7.02; 1.86 (2; 10)	6.06; 2.25 (2; 10)	7.00; 1.70 (2; 10)

M, mean; SD, standard deviation; Certainty, certainty of being infected by COVID-19; Ep, extroversion; Ap, agreeableness; Cp, conscientiousness; Np, neuroticism; and Op, openness personality dimensions.

**Table 2:** Distribution of participants based on the certainty of being infected by COVID-19.

Certainty of being infected	Frequency	Percent	Cumulative percent
Sure not infected	101	26.9	26.9
Probably not infected	171	45.6	72.5
Uncertain	67	17.9	90.4
Quite certain	28	7.5	97.9
Certain	8	2.1	100
Total	375	100	

#### Correlations

Certainty of being infected by COVID-19 correlated with health anxiety and conscientiousness. Age was positively correlated with conscientiousness and negatively correlated with health anxiety and neuroticism. Health anxiety was negatively correlated with agreeableness, conscientiousness and positively correlated with neuroticism. Moreover, neuroticism was negatively correlated with conscientiousness (Table 3).

**Table 3:** The correlations between study variables.

S. No	1	2	3	4	5	6	7	8
1. Certainty	1							
2. Age	−0.03	1						
3. Health Anxiety	0.22^**^	−0.21^*^	1					
4. Extraversion	0.03	0.02	−0.06	1				
5. Agreeableness	−0.02	0.01	−0.18^*^	0.1	1			
6. Conscientiousness	0.12^**^	0.24^**^	−0.18^**^	0.15^**^	0.15^**^	1		
7. Neuroticism	0.09	−0.17^*^	0.41^**^	−0.1	−0.22^**^	−0.20^**^	1	
8. Openness	−0.01	0.1	0.07	0.03	0.12^*^	0.09	0.06	1

N for all variables = 375.

* *p* < 0.05;

** *p* < 0.01.

### Regression Analysis

The results of the multiple regression showed that health anxiety [*B* = 0.04, *β* = 0.24, *S.E* (HC) = 0.01, *p* = 0.0001], and conscientiousness [*B* = 0.08, *β* = 0.16, *S.E* (HC) = 03, *p* = 0.006] were significant predictors of certainty of being infected by COVID-19 [*R* = 0.29, *R* ^2^ = 0.0827, *F*(8,366) = 3.23, *p* = 0.001]. No other variable was shown as a significant predictor of certainty of being infected by COVID-19. The model explained 29% of variance of certainty of being infected by COVID-19 (Table 4).

**Table 4:** The characteristics of the multiple regression results.

Predictors	B	β	*SE* (HC)
Age	0.00	0.02	0.00
Sex	0.14	0.07	0.10
Health anxiety	0.04^***^	0.24^***^	0.01
Extraversion	0.02	0.03	0.02
Agreeableness	0.01	0.02	0.03
Conscientiousness	0.08^**^	0.16^**^	0.03
Neuroticism	0.02	0.04	0.02
Openness	0.01	−0.04	0.02
*R* ^2^ (*Root MSE*)	0.08 (0.93)		
*F* (*df*)	3.23^***^ (8, 366)		

The dependent variable was certainty of being infected by COVID-19. B: coefficients. β: Standardized Beta coefficients. SE (HC): heteroscedasticity-consistent SEs. Root MSE: root mean square errors.

** *p* < 0.01;

*** *p* < 0.001.

## Discussion

The results of the present study showed that certainty of being infected by COVID-19 was predicted by the personality dimension conscientiousness and the cognitive-emotive factor health anxiety. This model explained 29% of variance in certainty of being infected by COVID-19. Sex, age, and other personality dimensions such as neuroticism did not emerge as significant predictors for certainty of being infected by COVID-19. Moreover, conscientiousness and health anxiety were negatively correlated.

We previously showed that certainty of being infected acted as a nocebo and exacerbated the perception of symptoms similar to COVID-19 (Daniali and Flaten, 2021, under review). This means that individuals who were more certain about being infected by COVID-19, were more likely to report, e.g., a sore throat or headache as COVID-19 symptoms. The current findings highlight the contribution of health anxiety and conscientiousness in such a nocebo belief.

Higher conscientiousness led to stronger beliefs of being infected by COVID-19. Former studies have shown that conscientious individuals tend to expose themselves to more COVID-19 news, more strictly follow the preventive health advice such as keeping a good hand hygiene (Carvalho et al., 2020), and practice precautionary behaviors such as stockpiling of toilet papers (Garbe et al., 2020). Along with the same line, it can be assumed that the stronger belief in being infected by COVID-19 in conscientious individuals can be due to an increased attention toward symptoms that resemble COVID-19 symptoms. However, this assumption requires more investigation, as to our knowledge, our finding is the first to demonstrate the effects of conscientiousness on health-related beliefs. Conscientiousness is known to be associated with being disciplined, rule-following, and self-controlled (Costa and McCrae, 1987, 1992); the negative correlation between conscientiousness with neuroticism here partially supports this notion.

Higher health anxiety also led to a stronger belief of being infected by COVID-19. This fits well with findings that health anxiety predicts hypochondriasis (e.g., Bleichhardt and Hiller, 2007; Faasse and Petrie, 2013; Jungmann and Witthöft, 2020). Individuals who are highly worried about their health status, tend to misinterpret bodily experiences as indications of having caught a disease. Like above, such association may be explained through an inclination to over-contemplate about the disease and its potential catastrophic consequences (e.g., Faasse and Petrie, 2013). Health anxiety is associated with emotional instability and overthinking about health-related negative consequences (e.g., Ferguson, 2009); this is evidenced here by the positive correlation of health anxiety with neuroticism.

Although both conscientiousness and health anxiety predicted higher certainty of being infected by COVID-19, the factors were negatively correlated, as shown in prior studies (Nikčević et al., 2020). Thus, there seems to be several ways in which different individuals may develop similar health-related beliefs. The literature on the psychological processes that underlie the formation of beliefs is scarce. However, as beliefs or expectations are central concepts in the elicitation and amplitude of placebo and nocebo effects, the development and structure of beliefs will be studied further.

Contrary to our proposed model, neuroticism failed to predict certainty of being infected by COVID-19, suggesting that certainty of being infected by COVID-19 is not impacted by being constantly anxious and experiencing negative affect. This is consistent with prior studies that found no association between personality traits and expectations of higher pain. For instance, Aslaksen and Lyby (2015) studied the effects of personality traits and fear of pain on nocebo hyperalgesia (i.e., increase in pain due to an inert agent) and reported that no personality trait was significantly associated with the nocebo effect.

Our analyses did not reveal a specific contribution for participants’ age on the relationship between the personality traits, health anxiety, and certainty of being infected by COVID-19. There is no consensus yet on how age and sex can moderate the effects of personality factors on negative psychological consequences related to COVID-19. For example, Aschwanden et al. (2020) found that age moderated the association between personality traits and reactions toward COVID-19, as being elder was associated with stronger personality-reflected behaviors toward COVID-19 such as higher neuroticism reflected through being more concerned about COVID-19, or conscientiousness reflected through more precautionary behaviors. However, Nikčević et al. (2020) found no effects for participants’ age on the association between personality traits, health anxiety, and negative emotions related to COVID-19.

Finally, participant sex did not emerge as a significant predictor of certainty of being infected by COVID-19, suggesting that this nocebo belief occur in both sexes. This result might have been due to the unequal numbers of males (*N* = 126) to females (*N* = 249). However, in the first study (Daniali and Flaten, 2021, under review) females reported higher COVID-like symptoms compared to males (see also review by Vambheim and Flaten, 2017). This notion suggests that even though the belief of being infected by COVID-19 may not differ across sexes, the nocebo effect still seems to be higher in females. Thus, even if beliefs are similar in males and females, the nocebo effect stemming from these beliefs seems to be stronger in females. This could be due to a response bias as males often under-report pain and associated emotions (e.g., Aslaksen et al., 2007), or to psychophysiological processes associated with placebo and nocebo responses. These hypotheses will be followed-up in future studies.

## Conclusion

This study showed that being conscientious and worried about health made individuals susceptible toward developing a belief of being infected by COVID-19. Such finding may have clinical implications, as individuals high in these traits may over-report COVID-like symptoms. In settings, where COVID-19 testing services are provided, over-report of symptoms may be expected from individuals who show high health concerns. Moreover, providing advice about the likelihood of misinterpreting symptoms similar to COVID-19 symptoms may be useful for individuals with high levels of conscientiousness. The findings also have theoretical implications in the understanding of psychological processes that lead to development of beliefs or expectations underlying placebo and nocebo effects.

## Recommendations for Future Studies

Prospective studies are recommended to consider the followings: firstly, this study showed that conscientiousness along health anxiety dispose individuals to develop a certainty of being infected by COVID-19. However, not much is known about the effects of personality traits on nocebo effects and still more investigations are warranted. Attempts to describe the extent to which an individual responds to placebo treatment through a single personality trait may be too limited. Thus, a transactional model of placebo responding, in which dispositional characteristics dynamically interact with environmental contingencies, has been proposed by Darragh et al. (2015). According to this model, the overlaps among the personality traits suggest that placebo responsiveness could be conceptualized in terms of a two-faceted construct consisting of an inward and an outward orientation. Therefore, it might be interesting for prospective studies to investigate if highly conscientious or health-concerned individuals can be placed within any of these two categories. Secondly, the applicability of this model to other populations needs to be investigated; for example, this is important to know whether such a model is confirmed for individuals who request for a COVID-19 test. Thirdly, based on the negative association between health anxiety and conscientiousness, there is a likelihood for a distinction between the quality or direction (i.e., positive or negative) of expectations based on health anxiety and expectations based on conscientiousness. It is of importance to investigate whether higher certainty of being infected that is influenced by higher health anxiety results in more negative outcomes; and contrastingly, whether certainty that is stemmed from high conscientiousness results in more preventive and constructive behaviors toward COVID-19. Fourthly, the effects of other contextual factors such as being constantly exposed to pandemic news (Gao et al., 2020), or the characteristics of the health care providers (Daniali and Flaten, 2019), and if those can mediate the influence of conscientiousness or health anxiety on certainty of being infected by COVID-19 needs to be investigated. Fifthly, whether such personality and/or cognitive constructs can lead to higher psychophysiological nocebo or placebo responses, such as higher blood pressure, as shown in Daniali and Flaten (2020), should be investigated in future studies. Moreover, how such a belief of being infected should interrupt the health guidelines and treatment procedures is of importance and requires investigation. Finally, there may be sex differences in how individuals react, subjectively and physiologically, to their health-related beliefs (Vambheim et al., 2021, under review). Taken together, the findings from the present study and that of Daniali and Flaten (2021, under review) show that even though when males and females have similar beliefs about being infected by COVID-19 or not, females report more symptoms, i.e., more nocebo effects.

## Limitations

Briefly, the methodological and procedural limitations of the present study include the followings: the sample was biased as most of the participants were highly educated and young with only a small proportion of respondents being over the age of 60. This may have affected the outcomes and therefore, caution is required when generalizing the findings. Also, as in this study, causation cannot be concluded from cross-sectional studies. There are also disadvantages for online studies, such as dishonest answers, fatigue effects, and reckless answering. Regarding other limitations, it should be first noted that no information was gathered about the country of participants, and the course of the pandemic was different across countries, and this can have affected the results. Secondly, certainty of being infected by COVID-19 was investigated using a single item. This may have resulted in less variability in the outcome variable, restricting the psychometric reliability and validity of the present findings. Finally, although only participants who were not tested for COVID-19 were included, it is still possible that some participants were COVID-19 positive.

## Data Availability Statement

The raw data supporting the conclusions of this article will be made available by the authors, without undue reservation.

## Ethics Statement

The studies involving human participants were reviewed and approved by the Regional Committee for Medical and Health Research Ethics, Norway (REK; project number: 142652), and the Norwegian Center for Research Data (NSD; project number: 605612). The participants provided their written informed consent to participate in this study.

## Author Contributions

HD and MF designed and conducted the study. Both authors had mutually collaborated on analyzing the data, drafting, revising and preparing the final manuscript.

### Conflict of Interest

The authors declare that the research was conducted in the absence of any commercial or financial relationships that could be construed as a potential conflict of interest.
